# Preparing for Parkinson’s disease prevention trials: Current progress and future directions

**DOI:** 10.1177/1877718X251334050

**Published:** 2025-04-27

**Authors:** Sarah Bouhadoun, Aline Delva, Michael A Schwarzschild, Ronald B Postuma

**Affiliations:** 1 The Neuro (Montreal Neurological Institute-Hospital), McGill University, Montreal, Canada; 2 Department of Neurology and Neurosurgery, McGill University, Montreal, Quebec, Canada; 3 Department of Neurology, Massachusetts General Hospital, Boston, MA, USA; 4 Harvard Medical School, Boston, MA, USA; 5 Centre for Advanced Research in Sleep Medicine, Hôpital du Sacré-Coeur de Montréal, Montreal, Canada; 6 Research Institute of McGill University Health Centre, Montreal, Canada

**Keywords:** Parkinson’s disease, neuroprotective agents, biomarkers, prodromal symptoms, alpha-synuclein, exercise, disease prevention, clinical trial

## Abstract

In recent decades, numerous clinical trials have aimed to delay or prevent Parkinson's disease (PD) progression. Despite the theoretical promise and encouraging preclinical data, none have shown clear efficacy in slowing or preventing PD progression, related to several key limitations. Conventional motor and non-motor scales often fall short in detecting early disease changes, while the heterogeneity of PD phenotypes complicates treatment efficacy. The timing of interventions is also critical, as most trials target patients already in advanced stages of neurodegeneration. A deeper understanding of the preclinical phase and the emergence of new pathological frameworks have shifted the focus toward preventing the onset of clinical PD. Recent advances in biomarker research, including tissue, fluid, and imaging markers, are poised to transform PD research by improving patient selection, stratification, and disease progression monitoring. New biologically grounded frameworks for classifying synucleinopathies aim to distinguish biological subtypes from clinical phenotypes, enabling more targeted prevention trials. Successful PD prevention trials will require early enrollment of individuals at the highest risk, employing low-risk personalized interventions, with biomarkers or sensitive clinical markers as endpoints. Early involvement of key stakeholders will be essential to ensure that trials are timely, ethically sound, and aligned with the needs of the PD community.

## Plain language summary

In recent decades, many clinical trials have attempted to delay or prevent the progression of Parkinson's disease (PD), but none have demonstrated clear efficacy in achieving this goal. However, with a deeper understanding of the pre-motor phase and the development of new neurobiological models, research has increasingly shifted towards PD prevention. Advances in various types of biomarkers, such as blood and imaging markers, are expected to enhance PD research by improving participant selection and providing more accurate measures of disease progression. Early engagement of key stakeholders will be crucial to ensure that trials address the needs of the PD community.

## Part I: traditional neuroprotection trials

Numerous trials have explored potential neuroprotective interventions for Parkinson's disease (PD), targeting neuroprotective pathways, pathophysiological mechanisms, or genetic factors. Early efforts focused on MAO-B inhibitors, Coenzyme Q10, creatine, vitamin E, urate (inosine), and isradipine, all hypothesized to protect dopaminergic neurons or reduce oxidative stress, but ultimately, none demonstrated clear disease-modifying effects.^1–8^ Similarly, epidemiological associations led to trials investigating caffeine and nicotine, though results remain inconclusive.^9–12^

More recent efforts have shifted toward novel therapeutic targets. Glucagon-like peptide-1 receptor agonists (GLP-1RAs) showed neuroprotective potential in preclinical models, but clinical trials yielded mixed results; the LIXIPARK trial suggested modest motor benefit, while the PRISM trial (NLY01) found no significant effects, though post-hoc analyses hinted at a possible response in younger patients.^13,14^

Monoclonal antibodies targeting alpha-synuclein aggregates have also been investigated. The PASADENA (prasinezumab) and SPARK (cinpanemab) trials found no significant differences in MDS-UPDRS progression, though prasinezumab showed potential benefits on MDS-UPDRS Part III and digital biomarkers, particularly in rapidly progressing subtypes. Follow-up data from PASADENA also suggested slower disease progression compared to natural history controls.^15–18^ Peer-reviewed results and conclusions of the PADOVA trials (prasinezumab phase IIb trial) are expected in 2025; however, the first release of results concluded that the primary endpoint was missed but some data suggest possible benefit in early-stage PD.^19^

Ongoing phase 3 trials continue to explore new and repurposed agents. Exenatide-PD3, investigating another GLP-1RA, recently announced negative primary outcome results. Meanwhile, the ASPro-PD and GREAT trials are assessing ambroxol, which may enhance glucocerebrosidase (GCase) activity, with primary outcomes focused on motor progression and secondary endpoints including neuroimaging and biomarker analyses.^20,21^

Given the limited success of pharmacological interventions, interest has grown in non-pharmacological strategies, particularly exercise. Studies such as Park-in-Shape and SPARX demonstrated that structured aerobic exercise may stabilize or slow motor symptom progression in early PD. SPARX3 is currently evaluating long-term effects with neuroimaging to determine whether exercise provides symptomatic, plasticity-modifying, or truly neuroprotective benefits.^22–27^ Other lifestyle interventions, such as dietary modifications, remain under investigation. While observational studies suggest potential benefits of the Mediterranean and MIND diets in reducing PD risk and progression, large-scale interventional trials are lacking.^28,29^

## Part II: understanding the limitations of previous PD trials

Despite the theoretical promise of several of these interventions, largely supported by preclinical data, none have succeeded in demonstrating clear efficacy in preventing or slowing the progression of PD. However, despite their neutral efficacy, these trials have been successful in advancing prospects for demonstrating positive results in future RCTs and enhancing our understanding of targets to prioritize. The disconnect between promising preclinical findings and the lack of successful translation into effective clinical therapies remains a substantial challenge. A closer analysis of these trials highlights several potential factors that may explain the observed shortcomings.

The limited availability and use of reliable biomarkers may have made more subtle effects impossible to observe and limited the ability to ‘fail early’ when evaluating potential targets. The reliance on traditional motor and non-motor scales, such as the MDS-UPDRS, though valuable, often falls short in detecting subtle and nuanced changes, particularly in early patients and with shorter durations of exposure. These scales also suffer from limitations like inter-rater variability and floor or ceiling effects, which can further diminish their effectiveness.

Another major challenge is disease heterogeneity, as the presence of several distinct subtypes and phenotypes among PD participants adds a layer of complexity to these efforts.^30^ Because treatments effective for one subgroup may not benefit others, achieving overall efficacy across such a heterogeneous patient population is particularly challenging. One crucial factor that may affect treatment efficacy in PD trials is Alzheimer's disease related co-pathology, which is increasingly recognized as a significant contributor to cognitive decline and disease progression in PD.^31–33^ Alzheimer's disease pathological hallmarks, including amyloid-β plaques and tau neurofibrillary tangles, are frequently found in post-mortem PD brains, particularly in those with cognitive impairment, suggesting that many PD patients have mixed pathologies rather than a pure synucleinopathy.^34–36^ Different studies have also shown that lower baseline cerebrospinal fluid (CSF) amyloid-β42 levels correlate with faster declines in cognitive, autonomic, and motor function in early PD.^37–39^ Moreover, genetic risk factors for Alzheimer's disease, including *APOE* ε4, have been associated with greater cognitive deterioration in PD, further supporting a shared pathological mechanism.^40,41^

The presence of co-pathologies may reduce the effectiveness of highly targeted disease-modifying therapies, for example monoclonal antibodies against α-synuclein. If clinical trials enroll PD patients with significant Alzheimer's disease co-pathology, therapeutic effects may be underestimated, as amyloid and tau-driven neurodegeneration may progress independently of α-synuclein-targeting therapies. Incorporating biomarkers for Alzheimer's disease pathology, such as plasma pTau217, CSF markers or amyloid PET imaging, into trial screening could help ensure that trials focus on PD patients who are most likely to benefit from synuclein-targeting therapies.

Finally, one of the most crucial limitations of these trials may be the timing of the intervention. Indeed, most focus on individuals already diagnosed with PD, at which point significant and irreversible neurodegeneration has likely occurred, reducing the potential effectiveness of the interventions studied. As a result, these trials primarily aim to slow the progression of traditionally diagnosed PD rather than preventing its onset. The deepened understanding of PD's extended preclinical/prodromal period over the past three decades, along with insights into its complex pathophysiological processes, has laid a strong foundation for the initiation of prevention trials. Indeed, by the early 1990s, the idea that PD may have a prolonged preclinical phase began to gain significant traction within the scientific community.^42,43^ This phase, marked by the onset of neurodegeneration in the absence of the hallmark motor symptoms, indicates that the pathological processes underlying PD could commence long before a clinical diagnosis rooted in motor signs was feasible.^44,45^ Although various non-motor and subtle motor symptoms may appear years, or even decades, before the emergence of overt motor signs,^46–48^ the identification of reliable biomarkers to detect these early stages was still largely speculative at that time.

In 2003, the introduction of the Braak classification marked a pivotal attempt to provide a biomarker-based neuropathological framework for early PD. This framework sought to map the sequential progression of α-synuclein pathology in PD and related synucleinopathies, tracing its putative spread from an enteric entry route towards the olfactory bulb, lower brainstem and neocortex.^49^ Since then, alternative hypotheses have been proposed, suggesting that Lewy body pathology might originate within the brain itself, possibly through an isolated olfactory route followed by cerebral propagation.^50,51^ A more recent model introduced a “brain-first” versus “body-first” dichotomy, which integrates aspects of these theories.^52–54^

With our enhanced understanding of the preclinical and prodromal phase of the disease and the development of new pathological frameworks, there is now an opportunity to shift the focus of trials toward preventing the onset of clinical PD rather than merely slowing its progression.

## Part III: recent advances in PD research and their implications for prevention trials

Our growing understanding of the pathophysiology of synucleinopathies has fueled significant advances and discoveries and profoundly impacted the design and implementation of prevention trials. One of the most pivotal recent breakthroughs has been the identification of biomarkers. In particular, the recent emergence of synuclein biomarkers has the potential to transform the design and execution of future clinical trials. Furthermore, these biomarker developments have led to the proposal of new biological definitions, diagnosis criteria and classification systems for PD,^55,56^ which could serve as a valuable framework in clinical research. In the context of clinical trials, tissue, fluid or imaging biomarkers can enhance patient selection and stratification, serve as objective measure of disease progression or assess target engagement.

### Recent advances in biomarkers

#### Tissue and fluid biomarkers: synuclein, neurofilament light chain and Alzheimer's disease related markers

Major progression has been made in the development of synuclein markers.^57,58^ The primary advances have been in the identification of synuclein seeding assays from biofluids, and the area of tissue biopsy (either with synuclein immunohistochemistry or synuclein seeding assays). For the first time, it is possible to detect synucleinopathy in living individuals with high sensitivity and specificity.^58–67^ Furthermore, these markers have the potential to enable the detection of synucleinopathy before symptoms of a manifest neurodegenerative disorder arise, facilitating earlier patient stratification and enrichment in clinical trials. Synuclein seeding assays (syn-SAA) have been extensively studied across various tissue types, demonstrating their capability to detect synucleinopathy in both prodromal and manifest stages of PD.^58–67^ Synuclein seeding assays (syn-SAA) in CSF have shown excellent sensitivity (>85%) and specificity (>90%) to detect α-synuclein seeding activity in Lewy body diseases including PD.^59–63,68^ In idiopathic/isolated REM-sleep behavior disorder (iRBD), an incipient synucleinopathy, sensitivity can be as high as 90%, depending on the cohort.^61,63,69^ This could be useful to enrich cohorts with participants having biomarker-proven prodromal synucleinopathy.

Moreover, CSF syn-SAA has shown potential in distinguishing between patients with PD or MSA, and showed a lower yield in LRRK2 carriers compared to sporadic PD, corroborating underlying pathophysiological differences.^59,61,70,71^ These results suggest that syn-SAA assays might be useful to subtype parkinsonism based on specific pathophysiological mechanisms. Given the invasiveness of CSF sampling, alternative tissues, such as skin and blood, have been explored to detect pathological α-synuclein. These more accessible and minimally invasive options have shown promising results, and would be easier to scale for broader clinical use compared to CSF.^64,72–74^ Nevertheless, further validation is needed before large-scale implementation in prevention trials is possible. Currently, seeding assays generally result in dichotomous outcome of ‘positive’ or ‘negative’, i.e., no quantitative α-synuclein outcome measure is available. This hampers the potential use of syn-SAA markers to assess potential associations between synuclein concentrations and clinical severity or disease progression, or as outcome measures in clinical trials. L1CAM-positive extracellular vesicles (L1EV) have recently emerged as a promising approach for quantitative blood-based measurements of α-synuclein, offering potential for more nuanced assessments in future studies.^74,75^

In addition to synuclein, other biomarkers hold promise for use in preventive clinical trials. Neurofilament light chain (NfL) is a nonspecific marker for neurodegeneration and can be measured in plasma and CSF.^76–83^ NfL may be valuable for patient selection, especially in the differentiation between PD and atypical parkinsonism, as the latter show much higher levels than PD.^78,84^ As higher CSF or plasma NfL have been associated with baseline and/or longitudinal cognitive decline, study cohorts could be enriched with people at risk for future cognitive decline in PD.^76,77,79,80,83^ More recently, glial fibrillary acidic protein (GFAP) has emerged as a promising marker in PD. Some studies showed higher plasma or CSF GFAP levels in PD compared to controls, whereas other studies could not confirm this.^80,83^ Higher CSF GFAP has been associated with longitudinal cognitive decline and risk of dementia in PD.^80,83,85^ Further research is needed to elucidate the potential value of GFAP in PD. In the context of cognitive impairment and dementia in PD, markers of Alzheimer's disease pathology could be valuable, particularly with the advent of plasma-based biomarkers.^38,76,83,86–88^ Plasma pTau181, for example, has been shown to be elevated in PD compared to controls, and is suggested to hold more promise as a diagnostic and progression marker compared to Aβ_42/40_.^76,83,87^

#### Imaging biomarkers: brain MRI, PET and SPECT imaging

Beyond tissue and fluid biomarkers, substantial research has been conducted on structural and functional imaging biomarkers. Unlike tissue and fluid biomarkers, imaging biomarkers additionally provide regional information, which can offer further insights in spreading of the disease pathology, regional target engagement of specific therapeutics, measurement and quantification of treatment effects, and is important for the detection of potential side effects related to the intervention such as inflammation, edema or hemorrhage.

Brain MRI, a widely available and minimally invasive imaging tool, offers significant potential as a biomarker in PD. MRI can detect cortical and subcortical brain atrophy in de novo patients and even those with prodromal disease, which often correlates with early cognitive decline.^89,90^ Other more specific MRI sequences have also shown encouraging results, even for use as progression markers.^91^ For instance, T1-weighted neuromelanin-sensitive MRI has revealed reduced signal intensity or volume of substantia nigra (SN) and/or locus coeruleus in PD with high accuracy, showing progression from the prodromal iRBD stage to more advanced disease stages, confined within a specific spatiotemporal pattern.^91–97^ The nigrosomes are key structures within the SN, containing clusters of dopaminergic neurons.^98^ In PD, nigrosome degeneration follows a characteristic temporospatial pattern, with nigrosome-1 being affected first (which results in a ‘swallow-tail’ sign on MRI).^98^ Additional MRI-based susceptibility-sensitive contrasts have been studied to quantify nigrosome degeneration, including R2* relaxation rate mapping, susceptibility weighted imaging (SWI) and quantitative susceptibility mapping (QSM).^99^ Previous nigrosome imaging studies could detect early changes in PD with high accuracy, even in the prodromal stage, and imaging correlated with motor and non-motor symptoms in PD.^100–105^ Different susceptibility-sensitive contrasts may have different accuracy in early vs. later-stage PD, affecting their potential use as progression biomarker.^103,106^ Although nigrosome imaging modalities hold promise, they also face significant technical challenges, requiring complex processing and interpretation.^99,101^ Finally, diffusion MRI has shown that free water measurements in the substantia nigra increase from prodromal to early PD when compared to controls, correlating with more severe motor symptoms.^107–110^

Functional PET or SPECT imaging on the other hand has been shown suitable for use as a biomarker in PD but is generally considered more invasive and less readily accessible. Dopaminergic imaging deficits reflecting nigrostriatal degeneration—the hallmark of PD—demonstrates high diagnostic accuracy^111^; normal functional dopaminergic imaging effectively excludes PD according to the MDS diagnostic criteria and the recent neuronal synuclein disease integrated staging system.^45,56^ Nevertheless, functional dopaminergic imaging cannot reliably discriminate PD from dementia with Lewy bodies or atypical parkinsonism.^111^ Longitudinal dopamine transporter (DAT) imaging studies using ¹²³I-FP-CIT SPECT, and more recently ¹¹C-PE2I or ^18^F-PE2I PET, show progressive decline in DAT binding over time in PD,^112–117^ as well as in iRBD,^118,119^ supporting potential as a progression marker. Nevertheless, correlation with symptom and motor sign progression is incomplete, suggesting that DAT imaging may not be a suitable surrogate endpoint for clinical progression.

Recent research suggests that molecular SPECT imaging of cardiac sympathetic innervation with the norepinephrine analog [^123^I]MIBG might be complementary with dopaminergic imaging in the discrimination of brain- vs. body-first PD.^52^ Indeed, in prodromal or early PD, disease pathology may spread differently, making dopaminergic imaging and [^123^I]MIBG SPECT suitable biomarkers in different stages of the disease. For instance, in the body-first subtype, [^123^I]MIBG SPECT might show abnormalities early in the disease course, but could suffer from early floor effects whilst dopaminergic imaging could be more suitable as a disease progression biomarker, and vice versa for the brain-first subtype.

Another imaging modality is the quantification of brain metabolism using [^18^F]FDG PET. Pattern analysis of brain [^18^F]FDG PET has identified different metabolic patterns related to clinical syndromes in patients with PD, such as the PD motor-related pattern (PDRP),^120^ PD-related cognitive pattern (PDCP),^121^ and PD-related tremor pattern (PDTP).^122^ However, it has not been established that [^18^F]FDG PET could be easily applied at the individual level in PD patients, a potential limitation for trials.

### New PD classifications: NSD-ISS and SynNeurGe

These recent advances in biomarkers and validated in-vivo detection methods for neuronal alpha-synuclein (n-αsyn) have catalyzed a new direction in the classification of synucleinopathies, moving from a reliance on clinical features to a focus on their underlying biological substrates.^59,61,68,123–125^ This transition has led to the proposal of diagnostic and classification frameworks that incorporate the pathological hallmark of n-αsyn, alongside neurodegenerative and genetic factors specific to PD. Recently, the neuronal α-synuclein disease integrated staging system (NSD-ISS) and the SynNeurGe classification model have been suggested, aiming to provide a biologically grounded understanding and definition of these disorders.^55,56^ Although they differ on some critical details, the systems both rely upon two key components to classify sporadic PD, namely: 1) synuclein assays (CSF seeding assays only in the NSD, with SynNeurGe adding skin seeding and immunohistochemistry) and 2) documentation of neurodegeneration (dopamine imaging only in the NSD, with SynNeurGe adding MIBG scintigraphy and PD-networks on PET scans). These proposals, still perhaps early drafts, underscore the shift in focus to biologic definitions, help identify biologically distinct subtypes and facilitate both early therapeutic interventions and the design of targeted, personalized prevention trials. Of note, both systems make no distinction between prodromal PD and full clinical PD/DLB, recognizing that clinical manifestations emerge gradually and variably, and resist hard categorization.

The recent advances in our understanding of PD, including new definitions and classifications, biomarkers, and insights into disease progression, have brought us to a pivotal moment where the creation of optimal prevention trials is within reach. This progress prompts the crucial question: how should we design these trials for maximum effectiveness? Several reports have tackled this question, offering valuable insights and recommendations.^22,126–129^

## Part IV: How to create a prevention trial in PD?

**Figure 1.**
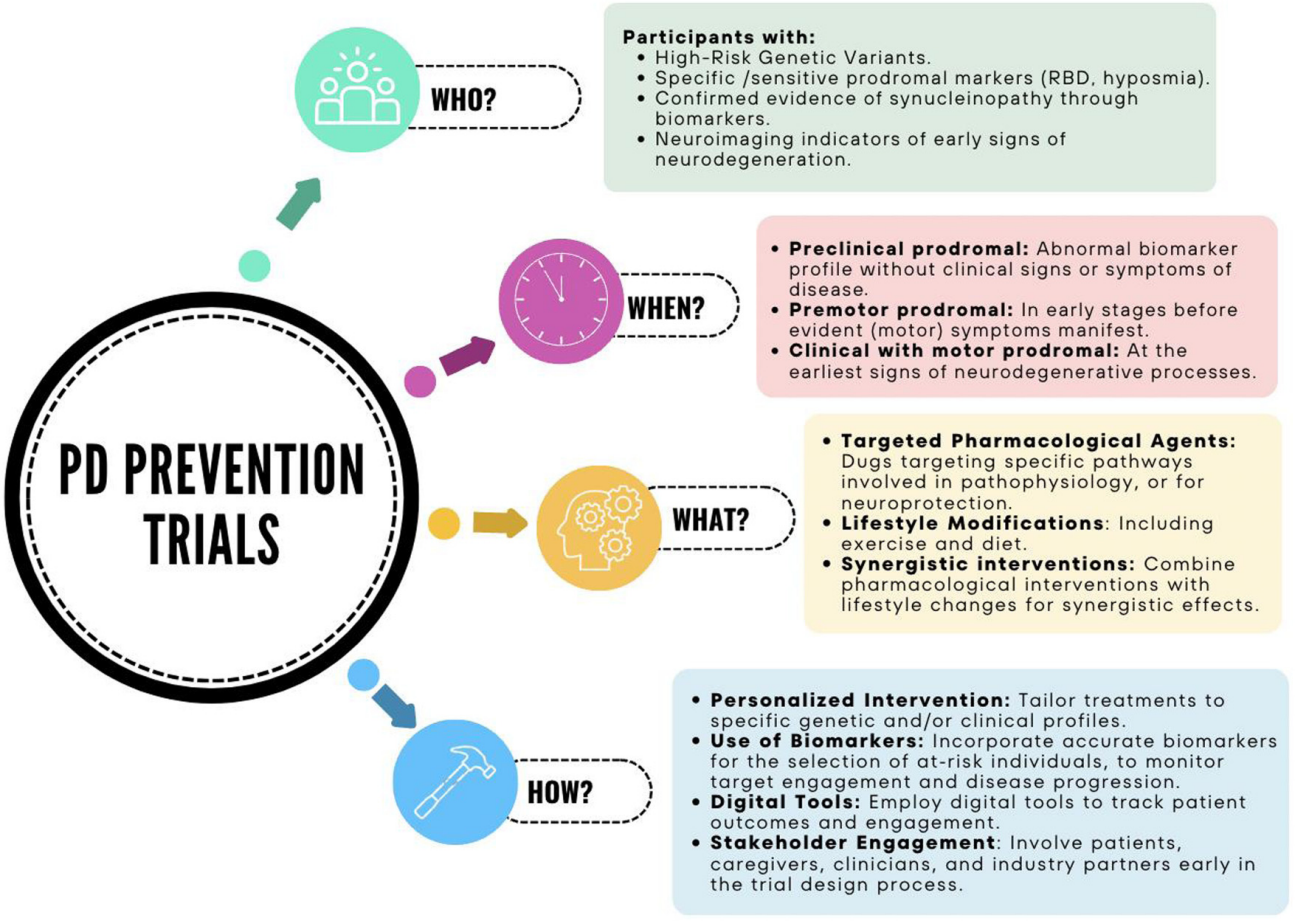
Framework for designing PD prevention trials. This framework outlines key components for developing effective Parkinson's disease prevention trials, including participant selection, optimal timing, targeted interventions, and methodologies for personalized treatment and biomarker integration.

Building on these insights, the development of successful prevention trials for PD requires a comprehensive approach that leverages advancements in molecular genetics, pathophysiology, and the identification of at-risk populations. Important questions remain, including which patients to include, which therapeutic agents to study and which primary outcomes to measure (Figure 1).

### Which patients to enroll?

To maximize the impact of these trials, it is ideal to enroll individuals in the earliest stage of PD, preferably before full manifestation of parkinsonism or cognitive impairment. Therefore, those with high-risk genetic variants such as LRRK2 or GBA, those exhibiting strong prodromal features like iRBD and hyposmia, and those with confirmed biological substrates of synucleinopathy are primary candidates.^46,47,130,131^

While clinical RBD cohorts have been a cornerstone of prodromal PD research, a biomarker-driven approach offers a precise and scalable strategy for trial enrollment, as illustrated by the inclusion of synuclein biomarkers and in recent PD classification frameworks. The high sensitivity and specificity of synuclein-based diagnostic markers (syn-SAA, tissue synuclein immunohistochemistry and tissue biopsy) for detecting early synucleinopathy can enable more targeted selection of at-risk individuals, rather than relying solely on clinical risk factors.

Despite these advantages, synuclein biomarkers have limitations. Their binary nature (positive/negative) precludes tracking progression, and potential reliance on CSF sampling for seeding assays makes large-scale screening less practical. While alternative biofluid and tissue-based synuclein assays (e.g., blood or skin) may improve accessibility, further validation is needed to establish their sensitivity and specificity in early-stage disease.^64,72–74^ Additionally, syn-SAA has shown lower detection rates in *LRRK2* carriers, which may necessitate subtype-specific enrollment strategies.^59,61,70,71^

Complementary biomarkers may further refine patient selection by identifying individuals at risk for more aggressive disease courses. NfL and GFAP could help stratify participants based on their likelihood of progression to motor or cognitive impairment.^76–83,85^ Similarly, Alzheimer's disease related markers, such as plasma pTau217, CSF markers or amyloid PET imaging, may assist in distinguishing individuals with co-pathology, allowing for stratification or exclusion from PD-specific prevention trials.^76,79,80,83,86,87^ However, while these biomarkers are well-established in Alzheimer's disease, their utility in identifying PD-related cognitive risk remains to be fully validated.

Ultimately, the success of PD prevention trials hinges on the ability to enroll individuals with biomarker-confirmed pathology before the emergence of overt symptoms. A multimodal approach integrating syn-SAA with complementary biomarkers and genetic or prodromal risk factors may offer the most effective strategy for optimizing cohort selection, ensuring that interventions are tested in populations with the highest potential for disease modification.

Another consideration for selecting patients for trial is inclusion of diverse cohorts of participants to improve generalizability of research results and conclusions. For example, currently, only a minority of patients with PD included in clinical research are non-white, and many clinical trial populations are considerably younger than the average PD patient. This has important implications for both patient enrollment strategies and the application of study results. Environmental and genetic risk factors vary widely across different populations, potentially leading to diverse disease manifestations and differential drug efficacy. For example, a recent genome-wide association study (GWAS) identified a new genetic risk factor in the GBA1 gene that is common in people of African ancestry but rare in non-African populations.^132^ This finding highlights the importance of balanced and diverse patient enrollment in future clinical research. Recognizing this need, different organizations representing key stakeholders are increasingly taking initiative to promote more inclusive research strategies.

### Which agents to use?

Priority would generally be given to low-risk interventions (participants in an early or prodromal stage do not feel sick, so may be less willing to tolerate bothersome side effects or invasive interventions). Repurposing drugs with well-established safety profiles, such as ibuprofen or caffeine, may offer a pragmatic and low-risk strategy for initial trials. Non-pharmacological or lifestyle interventions, which are generally well-tolerated, potentially in combination with well-tolerated pharmacological agents could yield synergistic effects, enhancing the overall efficacy of the interventions.^22^ Finally, it becomes especially critical to ensure that the participant is ‘on target’ for the intervention. This would include careful biomarker assessment as well as personalized interventions that are tailored to specific risk profiles. For instance, therapies aimed at the specific mechanisms linked to genetic variants are of primary interest.^127^ Agents targeting iRBD would wish to focus upon pathophysiologic mechanisms observed in that subpopulation; e.g., GBA (rather than LRRK2), synuclein, inflammation, etc.

### Which primary endpoint(s)?

Enrolling patients at earlier stages of disease also changes outcome measurement. Measures should capture meaningful clinical changes but also be sensitive enough to detect subtle disease change. Integrating biomarkers into trial design may represent the optimal method to monitor disease progression and therapeutic impact, providing a more sensitive and specific strategy than traditional motor and cognitive clinical assessment.^68^

Interest is growing in the potential of subtle non-motor symptoms as early indicators in PD research. For instance, olfactory function can decline more than 20 years before diagnosis, while impaired color vision may appear around 16 years prior, and constipation about 10 years before diagnosis; however, as they are very early markers, they tend not to progress, and so may be more suitable for selection criteria. Similarly, early motor symptoms also offer viable options for outcome measures in prevention trials. Indeed, the alternating tapping test shows deviations from normal values up to 12 years before phenoconversion, UPDRS II scores around 9 years prior, and the Purdue pegboard and time-up-and-go tests become abnormal 6–7 years before diagnosis.^46,48^ Unlike other markers, these show very good sensitivity to change. Quality of life and symptom burden metrics could provide another important dimension in assessing patient outcomes, although it is essential to recognize that functional impairment occurs late in the course of neurodegeneration, and therefore may be insufficiently sensitive to change.^133^ Finally, the use of digital metrics, including wearable devices and mobile health apps, can streamline the collection of these data, improve scalability and access, and may be particularly sensitive to change.

The early and active involvement of key stakeholders—including at-risk individuals, patients, clinicians, regulators, and industry partners—is essential to ensure that trials are relevant and timely, ethically sound and aligned with the needs of the PD community.

### Current ongoing prevention trials

Examples of early PD prevention trials among several recently or imminently enrolling^134^ include the Slow-SPEED-NL trial, a double-blind randomized controlled trial studying if volume and intensity of daily physical activity in iRBD can be increased over a 2-year period with a digital application. As secondary aims, the effect on prodromal motor and non-motor symptoms as well as on imaging biomarkers will be investigated, aiming to finally develop a composite score to estimate prodromal load. Furthermore, a recent collaboration between Parkinson's Progression Marker Initiative (PPMI) and Michael J. Fox Foundation launched the Path to Prevention Trial (P2P), a proof-of-concept phase 2 randomized clinical trial platform evaluating different investigational interventions in early neuronal synuclein disease using a single master protocol as framework. A trial of anti-inflammatory anti-TNF therapy with adalimumab for iRBD patients with hyposmia is currently in advanced planning stages to be run by the Parkinson Study Group. Many groups are collecting large populations of potential candidates, particularly iRBD patients; it appears that there are now sufficient numbers of iRBD patients to run several simultaneous preventative trials.

## Conclusion and future directions

The field of PD research has undergone transformative advances over the past decade, bringing us to a pivotal moment where the creation of optimal prevention trials is within reach. The comprehensive understanding of PD's prolonged preclinical and premotor phases, coupled with advances in biomarker discovery, genetic insights, and innovative therapeutic strategies, has laid a solid foundation for launching effective prevention trials. We are indeed ready to take the next steps.
